# The multifaceted roles of GSDME-mediated pyroptosis in cancer: therapeutic strategies and persisting obstacles

**DOI:** 10.1038/s41419-023-06382-y

**Published:** 2023-12-16

**Authors:** Yixiang Hu, Ya Liu, Lijuan Zong, Wenyou Zhang, Renzhu Liu, Qichang Xing, Zheng Liu, Qingzi Yan, Wencan Li, Haibo Lei, Xiang Liu

**Affiliations:** 1 Molecular Pharmacology Laboratory, Department of Clinical Pharmacy, Xiangtan Center Hospital, Xiangtan, 411100 China; 2 Honghao Zhou Research Institute, Xiangtan Center Hospital, Xiangtan, 411100 China; 3 Department of Rehabilitation Medicine, Zhongda Hospital of Southeast University, Nanjing, 210096 China; 4 Department of Pharmacy, Sir Run-Run Shaw Hospital, Zhejiang University School of Medicine, Hangzhou, 310006 China

**Keywords:** Cell death, Cancer immunotherapy

## Abstract

Pyroptosis is a novel regulated cell death (RCD) mode associated with inflammation and innate immunity. Gasdermin E (GSDME), a crucial component of the gasdermin (GSDM) family proteins, has the ability to convert caspase-3-mediated apoptosis to pyroptosis of cancer cells and activate anti-tumor immunity. Accumulating evidence indicates that GSDME methylation holds tremendous potential as a biomarker for early detection, diagnosis, prognosis, and treatment of tumors. In fact, GSDME-mediated pyroptosis performs a dual role in anti-tumor therapy. On the one side, pyroptotic cell death in tumors caused by GSDME contributes to inflammatory cytokines release, which transform the tumor immune microenvironment (TIME) from a ‘cold’ to a ‘hot’ state and significantly improve anti-tumor immunotherapy. However, due to GSDME is expressed in nearly all body tissues and immune cells, it can exacerbate chemotherapy toxicity and partially block immune response. How to achieve a balance between the two sides is a crucial research topic. Meanwhile, the potential functions of GSDME-mediated pyroptosis in anti-programmed cell death protein 1 (PD-1) therapy, antibody-drug conjugates (ADCs) therapy, and chimeric antigen receptor T cells (CAR-T cells) therapy have not yet been fully understood, and how to improve clinical outcomes persists obscure. In this review, we systematically summarize the latest research regarding the molecular mechanisms of pyroptosis and discuss the role of GSDME-mediated pyroptosis in anti-tumor immunity and its potential applications in cancer treatment.

## Facts

- GSDME-dependent pyroptotic pathways contribute to inflammatory tumor cell death and immune response activation.
- Assessing GSDME methylation levels is a beneficial strategy to detect cancer and distinguish between different tumor types.
- GSDME-mediated pyroptosis is responsible for certain chemotherapeutic medications’ toxicity and side effects.
- An in-depth understanding of the role of GSDME-mediated pyroptosis in TIME may provide novel strategies for improving anti-tumor immunotherapy.

## Open Questions

- What can be done to avoid adverse effects caused by GSDME-mediated pyroptosis?
- What if detecting GSDME expression levels could be a promising strategy to assess the risk and severity of CRS caused by CAR T-cell therapy?
- How do immune cells collaborate to inhibit or promote tumor progression via GSDME-mediated pyroptosis?
- How to find a balance point between the intervention of GSDME-mediated pyroptosis through inflammatory pathways and anti-tumor immunity?

## Introduction

Although tremendous advances have been made in anti-tumor therapeutic strategies over the past decade, cancer remains a pressing public health issue due to its rising incidence and mortality rates [1]. In 2020, the World Health Organization reported 19.3 million newly diagnosed cancer cases and approximately 10.0 million cancer-related deaths [2]. Conventional therapeutic approaches, such as chemotherapy, radiation therapy, and surgery, have been recognized to possess significant limitations [3]. Unfortunately, many patients with metastatic or recurrent diseases continue to experience unfavorable outcomes despite these treatments [4]. Studies have demonstrated that tumors are pathologically affected by oxidative stress, chronic inflammation, pathogen infection, immune destruction, and other factors [5–7]. Of note, the tumor immune microenvironment (TIME) is crucial for modulating tumor invasion, metastasis, and anti-tumor immunity [8]. Cancer treatment strategies rely heavily on inducing tumor cell death [4]. On the basis of cellular morphological characteristics, regulatory mechanisms, and biological functions, cell death can be classified into accidental cell death and regulated cell death (RCD) [9].

Pyroptosis is a type of RCD associated with inflammation. It is characterized by cell swelling, plasma membrane pores formation, intercellular content secretion, and proinflammatory effects [10]. This inflammatory cell death form is widely recognized as a biological mechanism crucial for maintaining organismal homeostasis [10]. As evidence accumulates, pyroptosis is distinguished by a faster occurrence and a more potent inflammatory response when compared to other RCD modes [11, 12]. Furthermore, pyroptosis is closely associated with inflammatory diseases caused by hypoxia, ER stress, pathogens, and tumors [13–16]. Importantly, pyroptosis plays a double-edged sword role in terms of reshaping the TIME. In one aspect, a mild inflammatory response caused by pyroptosis activates immune cells and eliminating tumor cells, maintaining the stability of the extracellular environment [10]. However, in the presence of prolonged and excessive pyroptosis effects, massive inflammatory and immune factors are released, causing normal cells to become cancerous [17].

Gasdermin (GSDM) family proteins, including GSDMs A–E, are key effectors that mediate pyroptosis [18]. In fact, pyroptosis is now recognized as an inflammatory form of RCD mediated by GSDMs [19]. In the past few years, GSDMD has been identified as the major executive protein for pyroptosis and depends on caspase-1/4/5/11 activation [20]. Recently, researchers have made noteworthy discoveries in the biological process of pyroptosis, revealing the crucial function of GSDME in the modulation of inflammation and immunology [21]. GSDME (also known as ICERE1 or DFNA5), which is present in both tumor cells and normal tissues, has the ability to convert caspase-3-mediated apoptosis to pyroptosis upon exposure to chemotherapy agents [22]. Caspase-3 was previously regarded as one of the primary apoptosis biomarkers. Interestingly, emerging evidence reveals that caspase-3 specifically splits GSDME at the linker region, thereby releasing the N-terminal domain and generate membrane holes [23]. This process leads to the release of interleukin (IL-1β/IL-18) and immunogenic molecules, disrupting ionic and water homeostasis, ultimately converting the RCD type from apoptosis to pyroptosis [23]. Furthermore, caspase-3 and GSDME-dependent pyroptotic pathways contribute to inflammatory tumor cell death and immune response activation, such as enhancing the anti-tumor activity of CD8^+^ T killer lymphocytes, macrophages, and tumor-infiltrating natural killer (NK) cells, which are intricately associated with anti-tumor immunity [21]. Very recently, however, it was also observed that GSDME gene methylation varied according to cancer type between tumor tissues and normal cells [24]. This indicates its potential as a detection biomarker for both pancancer and specific cancer types [24]. Overall, developing a more profound and enhanced knowledge of the role of GSDME-mediated pyroptosis in cancer may have substantial clinical utility as a diagnostic biological marker and provide novel strategies for preventing and treating tumors.

## Comparison molecular mechanisms of necroptosis, apoptosis, ferroptosis, cuproptosis, and pyroptosis

Cell death plays a critical role in multiple biological mechanisms, including maintaining normal homeostasis, eliminating harmful stimuli, and killing potentially neoplastic cells [25]. In recent years, it has been increasingly recognized that accidental cell death and RCD are the two principal forms of cell death [25]. The major types of RCD include pyroptosis, necroptosis, apoptosis, ferroptosis, and cuproptosis [26, 27]. Importantly, each RCD subroutine has its own initiator, effector, and executor, which also possess distinct histological and biochemical characteristics.

Necroptosis is a lytic inflammatory RCD mode characterized by cytoplasmic membrane disruption and cellular swelling [28]. Particularly, necroptosis is considered caspase-independent [28]. Instead, upon initiation by death receptors, necroptosis is mediated by receptor-interacting protein 1 (RIP1), RIP3, and mixed lineage kinase domain-like (MLKL), leading to MLKL pores formation [29]. Apoptosis is a non-inflammatory RCD mode. Histologically, apoptosis is characterized by condensation of chromatin, formation of apoptotic bodies, fragmentation of nuclei, shrinkage and pyknosis of cells [30]. Typically, the activation of apoptosis is initiated by non-inflammatory proteases, including caspase-3/7/9 [31]. Ferroptosis is a distinctive iron-dependent RCD triggered by excessive lipid peroxides accumulation in plasma membranes [32]. This molecular event is usually accompanied by intracellular iron overload and glutathione (GSH) exhaustion, resulting in reactive oxygen species (ROS) generation and plasma membrane rupture [33]. The latest research indicates that cuproptosis is a newly discovered form of RCD triggered by excess Cu^2+^ [34]. Particularly, elesclomol functions as an ionophore that promotes Cu^2+^ transport into cells [34]. Intracellular copper ions target and bind to lipoylated components in the tricarboxylic acid (TCA) cycle [35]. This process results in the instability of Fe-S cluster proteins, which ultimately causes proteotoxic stress and cell mortality [35].

Pyroptosis is an inflammatory form of RCD. The observation of pyroptosis dates back to 1992, when Zychlinsky and colleagues first noted this new form of cell death [36]. However, at the time, it was mistakenly identified as morphological changes in apoptosis mediated by caspase-1 in macrophages infected with Shigella flexneri [36]. A decade later, in 2001, this new caspase-1-dependent RCD was termed pyroptosis by Brennan [37]. Unlike other RCD forms, pyroptosis involves the innate immune system and is characterized by its ability to induce inflammation, recruit neutrophils, and modulate the immune response [13]. Due to several newly discovered molecular events, pyroptosis has recently received increased attention [38, 39]. During the early stage of pyroptosis, a distinct type of DNA damage occurs that sets it apart from apoptosis [30, 40]. Chromatin coagulation occurs in both apoptosis and pyroptosis [40]. However, in pyroptosis, the nucleus maintains intact and does not undergo karyorrhexis [41]. In contrast to necroptosis, apoptosis, and ferroptosis, pyroptosis is characterized by inflammasome formation and GSDMs-dependent cell membrane holes generation [30, 42]. Initially, pathogen-associated molecular patterns (PAMPs) and damage-associated molecular patterns (DAMPs) are recognized intracellularly by cytoplasmic pattern recognition receptors (PRRs), triggering the assembly of inflammasome complex and the maturity of inflammatory caspases [43]. After that, cells experience pores formation in the cytoplasmic membrane, followed by swelling and eventual lysis [44]. These pore-induced intracellular traps mediated by GSDMs eventually lead to the release of cell contents and pro-inflammatory mediators, including IL-1β/18 and DAMPs [19]. Taken together, the different characteristics between pyroptosis and other RCD modalities are briefly summarized in Table 1.

**Table 1:** Morphological characteristics and key biochemical pathway components of RCD pathways.

RCD types	Cellular morphological features	Key biochemical pathway components	Core characteristic (s)	Caspase-dependent	Pore-forming cause	Inflammation features	Immune features	Ref.
Necroptosis	Cytoplasmic swelling, pore formation on cells membranes, loss of plasma membrane integrity, swelling of cytoplasmic organelles, and moderate chromatin condensation.	RIPK1, RIPK3, TRADD, NMEO, caspase-8, and MLKL	Necrosome	√	MLKL-dependent pores	Inflammatory cell death	Immunogenic cell death	[30, 31]
Apoptosis	Chromatin condensation, plasma membrane blebbing, apoptotic body formation, nuclear fragmentation, cell shrinkage and pyknosis, and phagocytosis by neighboring cells.	Apaf-1, caspase-3/7/8/9, cytochrome c, Bid/tBid, BAX/BAK, BCL-2, and NF-κb	Apoptotic body	√	No	non-inflammatory	Immunogenic cell death or tolerogenic cell death	[32, 33, 42]
Ferroptosis	Cells swelling, pore formation on cells membranes, smaller mitochondria with decreased cristae, elevated mitochondrial membrane densities, and accumulation of lipid peroxides in plasma membranes	Fe^2+^, GPX4, ROS, PUFAs, ACSL4, LPCAT3, ALOXs, SLC40A1, TF-TFRC,	Iron accumulation and lipid peroxidation	×	Iron-dependent lipid peroxidation	Inflammatory cell death	Immunogenic cell death	[34, 35]
Cuproptosis	Mitochondrial shrinkage, mitochondrial membrane rupture, reduction in the mitochondrial crest and mitochondrial membrane lysis	Cu^+^/Cu^2+^, FDX1, GPX4, SLC31A1, LIAS, ROS, ATP7A/B, GSH	DLAT aggregation and loss of Fe-S cluster	×	No	Undetermined	Immunogenic cell death	[36, 37]
Pyroptosis	Cell swelling, membrane pores formation, membrane rupture, plasma membrane blebbing, moderately condensed chromatin	NLRP3 inflammasome, caspase-1/3/4/5/11, GSDMD, GSDME, IL-1β, and IL-18	Inflammasome	√	GSDMs-dependent pores	Inflammatory cell death	Immunogenic cell death	[14, 39–41]

## Overview of the biological functions of GSDME

The wild-type GSDME is composed of 10 exons which genetically encode a protein consisting of 496 amino acids with a molecular weight of 55 kDa [45]. It consists of an autoinhibitory C-terminal domain (GSDME-CT) and an N-terminal domain (GSDME-NT) connected by an interdomain linker. GSDME-NT has been found to initiate pyroptosis through its pore-forming activities [46]. Conversely, GSDME-CT provides cellular protection against pyroptosis under non-stimulating conditions [46].

GSDME is widely expressed in most tissues and organs [47]. In humans, the expression of GSDME is predominantly observed in the heart, brain, kidneys, and placenta [48–50]. The ability of GSDME to trigger cell death determines its crucial role in diseases. In 1998, GSDME was identified as a deafness gene implicated in the pathogenesis of hereditary hearing loss [51]. The mutations in GSDME resulted in transcriptional skipping of exon 8 and introduced a premature stop codon which destroyed GSDME-CT, leading to the loss of GSDME self-inhibitory activity and the emergence of cytotoxicity [52, 53]. However, the relationship between pyroptosis and hearing loss caused by the GSDME mutation remains uncertain. Moreover, GSDME has been linked to various disorders including atherosclerosis, kidney diseases, cystic fibrosis, and inflammatory skin diseases through genetic association studies [54–56]. Coincidentally, GSDME also plays a critical role in cancer. GSDME was identified as a potential tumor suppressor gene through genomic methylation monitoring. It functions as a transcriptional target of p53 and is inhibited in various types of cancer [57]. Evidence shows that the absence of GSDME renders certain chemotherapeutic drugs ineffective [58]. However, GSDME-mediated pyroptosis is also accountable for the toxicity and adverse effects of specific chemotherapeutic medications [22].

## The crosstalk between caspase-1, 4, 5, 11/GSDMD-mediated pyroptosis and caspase-3/GSDME-mediated pyroptosis

GSDMD has been extensively researched and is identified as a crucial gasdermin responsible for initiating pyroptosis [59]. Notably, GSDMD is a direct target of inflammatory caspases (caspase-1, 4, 5, and 11). In molecular terms, GSDMD-mediated pyroptosis can be classified into two distinct pathways: classical and non-classical pyroptotic pathways [60]. The classical pyroptotic pathway involves the recognition of DAMPs and PAMPs by Toll-like receptors (TLRs) and Nod-like receptors (NLRs) in response to intracellular and extracellular stimulation [61]. Upon activation of inflammasomes (particularly the NLRP3 inflammasome), caspase-1 is activated and GSDMD is cleaved to produce N-GSDMD fragments [62]. This leads to the formation of pores in the cell membrane, resulting in cell rupture and death. Additionally, cell contents such as lactate dehydrogenase (LDH) and inflammatory factors are released, exerting pro-inflammatory effects [62, 63]. In the non-classical pyroptotic pathway, caspase-4, 5, and 11 are involved instead of caspase-1. This pathway is initiated when Toll-like receptor 4 (TLR4) recognizes extracellular LPS [19, 64]. Once activated, caspase-4, 5, and 11 cleave GSDMD to generate GSDMD-NT and does not require inflammasome activation [65].

**Fig. 1:**
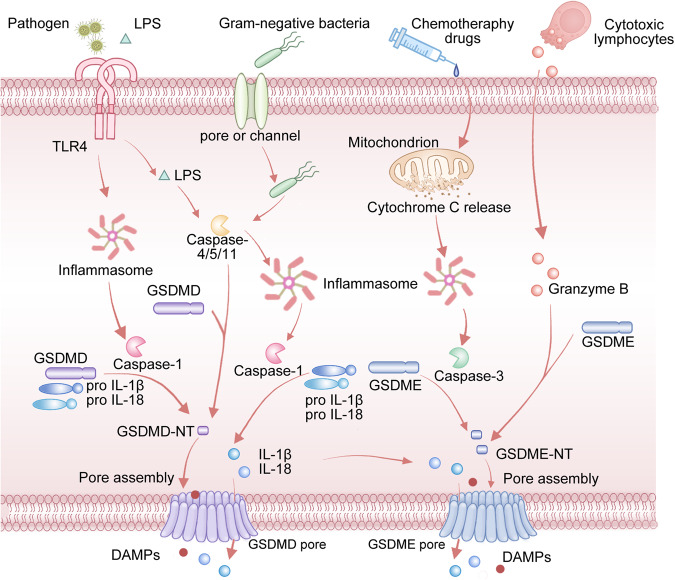
Graphical depiction of the mechanism of caspase-1, 4, 5, 11/GSDMD-mediated pyroptosis and caspase-3/GSDME-mediated pyroptosis. The following three pathways have been validated to trigger pyroptosis: caspase-1/GSDMD-mediated classical pyroptotic pathway, caspase-4/5/11/GSDMD-mediated non-classical pyroptotic pathway, and caspase-3/GSDME-mediated pyroptotic pathway. The classical pyroptotic pathway involves the recognition of exogenous and endogenous danger signals (e.g., pathogens). Upon NLRP3 inflammasome activation, caspase-1 is activated and GSDMD is cleaved to generate GSDMD-NT fragments. This molecular process results in membrane pores formation and cell lysis. In the non-classical pyroptotic pathway, caspase-4, 5, and 11 are involved. This pathway is initiated by extracellular LPS. Once activated, caspase-4, 5, and 11 cleave GSDMD to produce GSDMD-NT, which forms plasma membrane pores to induce cell death similar to the classical pyroptotic pathway. In the caspase-3/GSDME-mediated pyroptotic pathway, GSDME is activated by caspase-3 or granzyme B released by NK cells. Compared to GSDMD-mediated pyroptosis, GSDME-mediated pyroptosis doesn’t involve inflammasome assembly. This molecular event generates GSDME-NT and induces the formation of pyroptotic holes in cell membranes, resulting in cellular contents leakage. Notably, chemotherapy drugs could also elicit pyroptosis through the caspase-3/GSDME pathway.

In contrast to GSDMD, GSDME is activated by caspase-3 rather than caspases-1, 4, 5, and 11 [42, 50] (Fig. 1). Particularly, caspase-3 can be activated by apoptosis initiators caspase-8 and caspase-9 [66]. Furthermore, unlike GSDMD-mediated pyroptosis, GSDME-mediated pyroptosis does not require inflammasome assembly [67, 68]. After cutting by granzyme B released by NK cells or activated caspase-3 in the interdomain linker, the GSDME-NT domain is released to initiate the formation of pyroptotic pores in cell membranes, leading to cellular contents leakage and a subsequent proinflammatory effect [69]. Intriguingly, the level of GSDME is considered a crucial factor in caspase-3-mediated cell death form [23, 48]. When GSDME expression is low, cells are more likely to undergo apoptosis rather than pyroptosis in response to intrinsic stresses or extrinsic challenges [23].

## GSDME convert caspase-3-dependent apoptosis into pyroptosis

Apoptosis, unlike pyroptosis, is a type of RCD not associated with inflammation [38]. It is triggered by the activation of apoptotic caspases and can occur through either an intrinsic or extrinsic pathway [65]. The intrinsic pathway is triggered by multiple intracellular events (e.g., mitochondrial damage, endoplasmic reticulum stress, and ROS formation). These stimuli then contribute to the leakage of cytochrome c into the cytoplasm. Subsequently, after interacting with apoptotic protease activating factor-1 (Apaf-1), cytochrome c activates caspase-9. In turn, caspase-9 activates caspase-3/7, which ultimately results in cell death. The extrinsic pathway begins upon the oligomerization of cell surface death receptors. Once activated, pro-caspase-8 is recruited and converted to caspase-8, which then cleaves Bid to produce tBid and activates caspase-3/7, contributing to a series of molecular alterations and initiating apoptosis. In particular, apoptosis leads to rapid phagocytic clearance of dead or damaged cells, which is generally not immunogenic [70]. Apoptosis is also critical for inhibiting tumor growth, and certain anti-cancer medications work by inducing apoptosis [71]. The presence of an apoptosis-inducing domain in GSDME suggests that it may have an intrinsic ability to induce apoptosis. Transfection of mutant GSDME into mammalian cells resulted in cell apoptosis or exhibited toxic effects, whereas transfection of wildtype GSDME did not show such a phenomenon [72]. In fact, N-terminal domain found in both wildtype and mutant GSDME induces apoptosis and cell death. On the other hand, C-terminal domain can inhibit the apoptosis-inducing ability of N-terminal domain [73]. The exclusion of exon 8 can result in a shorter C-terminal domain for the mutant GSDME [73]. This can cause the release of autoinhibition activity and activate the N-terminal domain [73]. GSDME-NT appears to target mitochondria to promote cytochrome c release, thereby forming a self-sustaining feedback loop during apoptosis [31]. The available evidence suggests that mutations in GSDME lead to apoptosis through a series of interconnected pathways, including endoplasmic reticulum stress, mitochondrial damage, and oxidative stress [23, 74, 75].

**Fig. 2:**
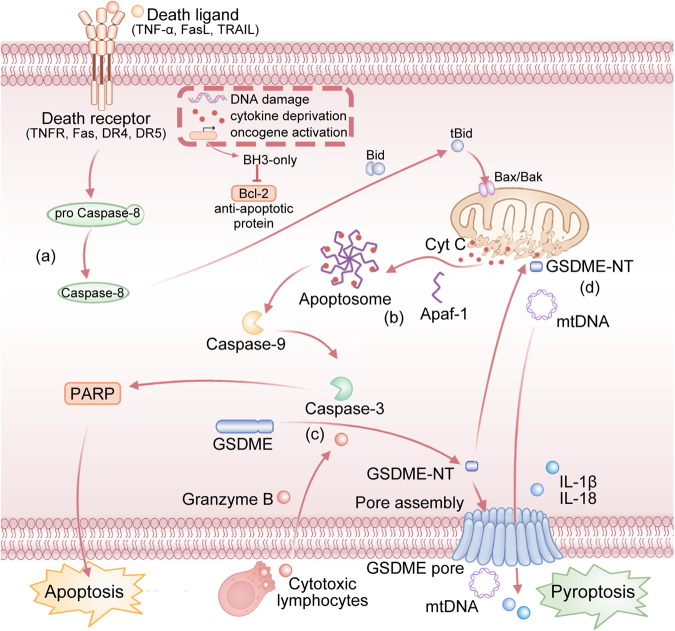
GSDME converts caspase-3-dependent apoptosis into pyroptosis. **a** The intrinsic apoptotic pathways. **b** The extrinsic apoptotic pathways. **c** The caspase-3/GSDME-mediated pyroptotic pathway. **d** GSDME-NT targets mitochondrial membranes to generate holes, causing the release of cytochrome c and mitochondrial DNA. GSDME works as a ‘transducer’ to determine whether a cell undergoes pyroptosis or apoptosis. Activated caspase-3 could cleave GSDME and induce pyroptotic cell death following the successful induction of apoptosis by caspase-3. Besides that, GSDME-NT cleaves the membrane of mitochondrial, which further triggers self-amplification of pyroptosis.

Caspase-3 executes functions by catalyzing the cleavage of peptide bonds following aspartic acid residues via the C-terminal cysteine residue [76]. Evidence suggests that caspase-3 acts as a crucial adaptor protein for caspase cascades [77]. It can be activated by intrinsic or extrinsic apoptotic pathways and is the only endogenous signal that triggers the cleavage of GSDME [78]. For decades, caspase-3 has been considered a key executor of apoptosis, but surprisingly, it can also be involved in pyroptosis in the presence of GSDME [23]. It was discovered that the absence of GSDME did not inhibit caspase-3-dependent cell death, but rather modified the form of cell death [22]. Actually, as the substrate of caspase-3, GSDME functions as a ‘transducer’ to determine whether a cell undergoes pyroptosis or apoptosis [79]. Rogers et al. discovered that activated caspase-3 could cleave GSDME and induce pyroptotic cell death following the successful induction of apoptosis by caspase-3 [31]. Additionally, GSDME-NT induces caspase-3 activation by cleaving the mitochondrial membrane, which further causes self-amplification of pyroptosis [80]. It is interesting to note that in cells with high GSDME expression, activated caspase-3 selectively cleaves GSDME to trigger pyroptosis before apoptosis, as pyroptosis is a faster process compared to apoptosis [23]. In cancer cell lines, caspase-3 can specifically cleave GSDME to induce a switch from apoptosis to pyroptosis, enhancing the sensitivity of cancer cells to chemotherapeutic agents [81, 82]. Further research found that in GSDME deletion cancer cells (e.g., Jurkat), chemotherapy drugs activate caspase-3, which cleaves the apoptotic substrate protein poly ADP-ribose polymerase (PARP) and leads to apoptosis [83]. However, in highly GSDME expressing cancer cells (e.g., SH-SY5Y), caspase-3 preferentially splits GSDME over PARP, resulting in caspase-3/GSDME-mediated pyroptosis [82]. Therefore, it can be concluded that GSDME is essential for the crosstalk between caspase-3-dependent apoptosis and pyroptosis (Fig. 2).

## GSDME methylation and its role in cancer detection

Biomarkers for clinical tumor detection must exhibit high stability, sensitivity, and specificity. Additionally, they should be capable of detecting cancer in its early stages. Ideal biomarkers can be obtained from solid tissues, urine, saliva, and, most importantly, circulating DNA in the bloodstream [84]. Accumulating evidences indicate that GSDME holds promise as a cancer detection biomarker [85]. GSDME is frequently observed as epigenetic silencing through DNA methylation in various cancers [49]. Consequently, assessing its methylation levels can serve as a valuable tool for detecting cancer and distinguishing between different tumor types [86].

The widespread methylation in promoter CpGs is correlated with transcriptional silencing and is a possible mechanism for inactivating tumor suppressor genes. Methylation of CpGs in the GSDME promoter was first observed in breast cancer [87]. KIM et al. performed TaqMan-methylation specific PCR of CpGs at GSDME promoter regions in 34 primary breast adenocarcinomas samples [87]. According to the results, a specific methylation of CpGs in GSDME promoter was identified associated with breast cancer [87]. In addition, a positive correlation was found between lymph node metastasis and the degree of GSDME methylation, suggesting that breast cancer patients with higher GSDME methylation levels are more likely to experience lymph node metastasis [87]. In patients with breast ductal adenocarcinoma, a negative correlation was observed between the degree of GSDME methylation and 5-year overall survival (OS), indicating that GSDME methylation is a prospective biomarker for determining prognosis [88]. Furthermore, GSDME expression is lower in estrogen receptor (ER)-positive tumors than in ER-negative tumors, providing evidence that ER may negatively regulate the expression of the GSDME gene via epigenetic modifications in the GSDME promoter [89]. Altogether, these findings strongly imply that GSDME methylation could be an excellent candidate for breast cancer screening and detection.

**Fig. 3:**
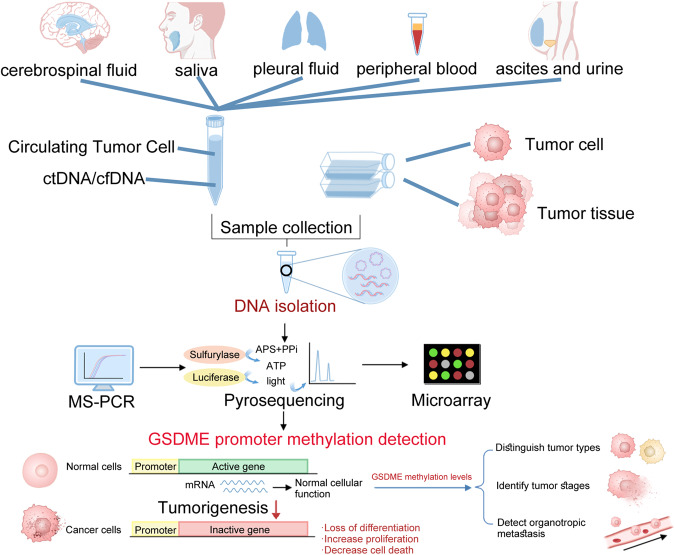
GSDME methylation and its role in cancer detection. Various body fluids (e.g., cerebrospinal fluid, saliva, pleural fluid, peripheral blood, ascites, and urine) and tumor tissue can be used to detect GSDME methylation. GSDME methylation may be a promising biomarker for distinguishing tumor types, identifying tumor stages, and detecting organotropic metastases.

In light of these findings, additional research has demonstrated that the potential of GSDME biomarkers is not restricted to breast cancer. Akino et al. discovered that GSDME promoter methylation can facilitate the transformation of normal gastric cells into malignant cells [90]. Inhibition of GSDME expression could accelerate gastric cancer development, confirming that GSDME methylation is crucial to the pathogenesis of gastric cancer and has the potential to serve as a diagnostic marker for this disease [91]. Studies conducted on colorectal cancers utilizing genome-wide methylation datasets from The Cancer Genome Atlas (TCGA) discovered that GSDME methylation could serve as a potential biomarker for detecting colorectal adenocarcinomas [92]. Furthermore, tumors with lymphatic vessel invasion and advanced tumor-node-metastasis (TNM) stage exhibited an increase in GSDME promoter methylation [92]. In a recent study, it was discovered that GSDME methylation is prevalent in various cancer types, with extensive hypermethylation in promoter and gene body CpGs [93]. According to these findings, GSDME methylation can be used as a pan-cancer biomarker and also help differentiate between different types of cancer [93]. Overview, GSDME methylation level is closely associated with the progression and organotropic metastasis of diverse types of tumors. Due to its exceptional expression and methylation properties in various cancers, GSDME is a promising gene candidate for future investigation as a tumor detection biomarker (Fig. 3).

## Role of GSDME-mediated pyroptosis in cancer therapy

**Fig. 4:**
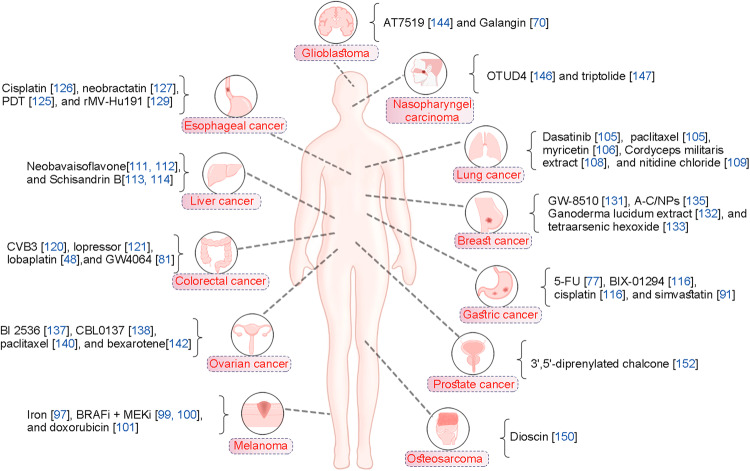
Potential anticancer therapeutic drugs targeting GSDME-mediated pyroptosis.

Tumorigenesis is a complex process influenced by multiple factors, such as the expression of proto- and anti-oncogenes, persistent inflammation, oxidative stress, and inflammatory TIME [94]. The elevated methylation of GSDME determines that tumor cells undergo pyroptosis rather than apoptosis, which indicates that combining methylase inhibitors and antineoplastic drugs may kill tumor cells more effectively [95]. Recent research has cast light on the crucial role of GSDME-mediated pyroptosis in treating various tumors, including melanoma, lung cancer, digestive malignancies, gynecologic malignancies, etc (Fig. 4).

### GSDME-mediated pyroptosis and melanoma

Melanoma is the primary cause of cutaneous malignancy-related mortality [96]. Recent research indicates that iron combined with ROS inducers carbonyl cyanide m-chlorophenyl hydrazone can effectively inhibit melanoma progression, with GSDME-mediated pyroptosis playing a critical role in this process [97]. In melanoma cells, abundant iron boosts ROS signaling, resulting in Tom20 oxidation and oligomerization [97]. Then, oxidized Tom20 recruits Bax to mitochondria, leading to cytochrome c release and caspase-3 activation, ultimately triggering GSDME-mediated pyroptosis [97]. Lage’s study found that GSDME expression elevation enhanced melanoma cells’ sensitivity to etoposide, indicating that GSDME-mediated pyroptosis contributes to melanoma etoposide resistance [98]. BRAF inhibitors (BRAFis) represent a significant advancement in melanoma treatment; however, their effectiveness is limited by rapid resistance emergence. Wang et al. demonstrated that IRF9-STAT2 signaling enhances adaptive resistance to BRAF inhibitors (BRAFis) in melanoma cells by regulating GSDME-mediated pyroptosis, thereby elucidating the pathogenesis of resistance in targeted therapy [99]. Similarly, BRAFis and MEK inhibitors (MEKis) have been observed to induce RCD in BRAF^V600E^-mutant melanoma cells [100]. Mechanistically, BRAFis + MEKis treatment activates caspase-3/GSDME signaling pathways, leading to tumor-associated T cells infiltration and activating dendritic cells to kill melanoma cells [100]. Eukaryotic elongation factor-2 kinase (eEF-2K) is a protein synthesis inhibitor that acts as a negative regulator and plays a vital role in malignant cell pyroptosis under diverse conditions [101]. Evidence confirmed that eEF-2K could amplify the pyroptosis-promoting impact of doxorubicin in melanoma cell lines expressing high levels of GSDME [101]. Very recently, another study confirmed that the GSDME-mediated pyroptosis is essential for initiating antibody recognition of intracellular antigens by inducing cell pyroptosis, inhibiting liver metastasis of circulating melanoma cells [102].

### GSDME-mediated pyroptosis and lung cancer

Lung cancer is the most ubiquitous and lethal malignancy worldwide [103]. Recent mechanistic work revealed that GSDME knockdown can trigger the transition from apoptosis-to-pyroptosis in lung cancer lines, confirming the hypothesis that GSDME expression determines the type of cell death in caspase-3-activated tumor cells [104]. In A549 cells, dasatinib can trigger pyroptosis and elevate GSDME levels in A549 cells without the involvement of p53 [105]. When GSDME expression is high, cisplatin and paclitaxel activate the caspase-3/GSDME pathway, convert normal chemotherapy-induced apoptosis into pyroptosis and inhibit lung cancer cell growth [105]. Han et al. revealed that myricetin could act as a pyroptosis agonist that activates caspase-3 via the ER stress pathway to facilitate GSDME cleavage and induce pyroptosis in lung cancer cells [106]. According to the latest research, GSDME inhibits EGFR signaling pathway and promotes non-small-cell lung cancer (NSCLC) cells’ survival by enhancing EGFR dimerization and activation [107]. In addition, GSDME interacts physically with EGFR, covering the EGFRY1045 site and contributing to its stability [107]. The hydrosoluble fraction of cordyceps militaris extract (CME), a dietary herb for lung cancer patients, has been shown to induce A549 cell death by simultaneously activating caspase-3/GSDME pyroptotic pathways and caspase-3/PARP apoptotic pathways [108]. Yu et al. revealed that the pyroptosis inhibitor (DSF) and apoptosis inhibitor (Z-VAD-FMK) prevented the cell mortality induced by nitidine chloride [109]. GSDME was cleaved in A549 and H1688 cells treated with nitidine chloride, accompanied increased caspase-3 cleavage [109]. Moreover, nitidine chloride substantially inhibited the PI3K/AKT pathway transduction and induced pyroptosis of tumor cells in vivo, indicating that nitidine chloride is a potential drug for lung cancer by inducing GSDME-dependent pyroptosis [109].

### GSDME-mediated pyroptosis and digestive malignancies

Liver cancer is one of the most prevalent and lethal digestive malignancies [110]. Neobavaisoflavone (NBIF), a natural active compound isolated from Psoralea, has anti-cancer and anti-inflammatory activities. A recent study revealed that hepatocellular carcinoma cells treated with NBIF exhibited pyroptotic characteristics [111]. Mechanistically, NBIF suppresses hepatocellular carcinoma cell proliferation by increasing the expression of Tom20 and triggering the caspase-3/GSDME pathway [112]. Schisandrin B has multiple therapeutic effects on liver disorders, such as repairing liver damage, reducing liver fibrosis, and inhibiting liver cancer [113]. Song et al. discovered that Schisandrin B causes HepG2 cells pyroptosis while working with NK cells through the activation of the perforin-granzyme B/caspase-3/GSDME pathway, indicating that Schisandrin B is a potential immunotherapy combination agent for liver cancer treatment [114].

Gastric cancer is a malignant tumor arising from the gastric mucosa’s epithelium and is the third leading cause of cancer-related mortality worldwide [115]. In 2007, epigenetic silencing of GSDME was initially discovered in primary gastric cancer [90]. Introducing GSDME into gastric cancer tumor cells inhibited their growth, indicating that GSDME has tumor suppressor activity [90]. Recent studies show that GSDME-expressed gastric cancer cells undergo pyroptosis when treated with chemotherapeutic agents such as 5-FU [77]. However, GSDME deletion by CRISPR-Cas9 converted 5-FU-induced pyroptosis to apoptosis in SGC-7901 cells, indicating that GSDME converts chemotherapy drug-induced apoptosis to pyroptosis in gastric cancer cells [77]. Another study confirmed that the combination of BIX-01294 and cisplatin could enhance the anti-cancer chemotherapy effect in gastric cancer cells by causing GSDME-mediated pyroptosis and activating autophagic flux [116]. This indicates that GSDME plays a crucial role in inducing tumor cells pyroptosis by chemotherapeutic agents in gastric cancer treatment. Furthermore, evidence suggests that GSDME has prognostic significance in gastric cancer. Therefore, GSDME’s capacity to suppress the proliferation of gastric cancer cells suggests that it can be utilized as a predictive biomarker [117]. Recently, Xia et al. found that simvastatin inhibits cell proliferation and induces caspase-3/GSDME-mediated pyroptosis to combat gastric malignancy [91]. Restoring GSDME expression with a DNA methyltransferase inhibitor can improve the susceptibility of gastric cancer cells to simvastatin [91].

Colorectal cancer remains the top 5 leading causes of tumor-related death in the digestive tract [118]. Studies have demonstrated that GSDME-mediated pyroptosis is also essential to the development of colorectal cancer [119]. Coxsackievirus group B3 (CVB3) is able to induce pyroptosis in colon cancer cell lines both in vivo and in vitro [120]. Mechanistically, CVB3-induced pyroptosis is promoted by ROS, which activates the caspase-3/GSDME pathway to produce pores in the plasma membrane [120]. These findings suggest that CVB3 is effective in treating colon cancer through the GSDME-mediated pyroptosis pathway. Similar to CVB3, lopressor can promote ROS phosphorylation and activate the caspase-3/GSDME axis to induce colon cancer cell pyroptosis [121]. Evidence shows that lobaplatin could trigger caspase-3 activation and GSDME cleavage, as well as activate the ROS/JNK/Bax mitochondrial apoptosis pathway, which increases cytochrome c release, indicating that GSDME-dependent pyroptosis is a potential mechanism by which loplatin eliminates colon cancer cells [48]. Synthetic FXR agonist GW4064 has the potential to exert a synergistic anticancer effect via the pyroptosis pathway [122]. Notably, GW4064 could enhance the anti-tumor effect of oxaliplatin against colorectal cancer [122]. Therefore, the combination of GW4064 and oxaliplatin may represent an alternative colorectal cancer treatment strategy. Recent studies have shown that the BAK/BAX and caspase-3/GSDME axis mediates chemotherapy-induced pyroptosis in colon cancer cells [81]. Importantly, 2-bromopalmitate (2-BP) inhibits GSDME palmitoylation during chemotherapy-induced pyroptosis [81].

Esophageal cancer is the sixth most prevalent form of cancer worldwide [123]. For the treatment of esophageal cancer, high resistance to radiotherapy and chemotherapy continues to be a significant obstacle [124]. Li et al. found that Photodynamic therapy (PDT) could induce pyroptosis in esophageal squamous cell carcinoma (ESCC) by modulating the PKM2/caspase-8/caspase-3/GSDME pathway, indicating that the clinical implementation of PDT in ESCC may have significant implications [125]. A recent study revealed that elevated STAT3β expression increases cisplatin sensitivity and promotes GSDME-mediated pyroptosis in ESCC cells after cisplatin exposure [126]. Mechanistically, STAT3β causes chemosensitivity in cisplatin-treated cells by disrupting mitochondrial electron chain transport and increasing ROS expression [126]. Another study revealed that Neobractatin (NBT) could inhibit the proliferation of esophageal cancer cells via GSDME-mediated pyroptosis [127]. According to mechanistic research, NBT contribute to ROS accumulation and active caspase-3 [127]. Recently, oncolytic viruses have been demonstrated to be an effective and promising option for cancer treatment [128]. in ESCC cells, rMV-Hu191 could induce mitochondrial dysfunction, which is mediated by BAK (BCL2 antagonist/killer 1) or BAX (BCL2 associated X) [129]. Further analysis discovered that rMV-Hu191 can effectively promote caspase-3/GSDME-mediated pyroptosis, which may increase oncolytic efficacy [129].

### GSDME-mediated pyroptosis and gynecologic malignancies

Breast cancer is the most prevalent malignancy among adult women [130]. Xu et al. provided preliminary evidence that GW-8510, an inhibitor of cyclin-dependent kinase (CDK) 2, could trigger GSDME-mediated pyroptosis in triple-negative breast cancer (TNBC) cells and mice models [131]. This mechanism may be associated with the potential of GW-8510 to enhance immune response and improve the efficacy of immunotherapy [131]. In a similar manner, Ganoderma lucidum extract can induce GSDME-mediated pyroptosis in TNBC cells and mouse models, while stimulating the peripheral immune system [132]. According to another study, tetraarsenic hexoxide-treated TNBC cells exhibited specific pyroptotic characteristics, inhibiting tumor formation and lung metastasis [133]. Mechanistically, tetraarsenic hexoxide significantly increased mitochondrial ROS production by preventing the phosphorylation of mitochondrial STAT3, thereby promoting GSDME-mediated pyroptosis in TNBC cells [133]. Evidence shows that the mitochondrial protein UCP1 could modulate TNBC cells proliferation and metastasis by inducing mitochondrial destruction to activate mitophagy and GSDME-mediated pyroptosis, suggesting that UCP1 may be a novel therapeutic target for TNBC [134]. Recently, Li et al. designed a carrier-free chemo-photodynamic nanoplatform (A-C/NPs) for treating breast cancer [135]. They discovered that A-C/NP treatment could trigger GSDME-mediated pyroptosis by disrupting mitochondrial homeostasis and ROS accumulation, resulting in immunogenic cell death and exhibiting potent cytotoxicity in breast cancer cells [135].

Ovarian cancer is one of the deadliest gynecological malignancies [136]. Huo et al. reported that BI 2536, a small molecule drug, effectively inhibited the proliferation of ovarian cancer cells by triggering both apoptosis and pyroptosis via the caspase-3/GSDME pathway [137]. Furthermore, BI 2536 promoted CD8^+^ T cell accumulation at tumor sites and exhibited anti-tumor activity. CBL0137 is a potential small molecule inhibitor that modulates p53 and nuclear factor-κB simultaneously. Recent evidence demonstrates that CBL0137 stimulates mitochondrial ROS production, BAX accumulation, and cyt c release to trigger caspase-3/GSDME-mediated pyroptosis in ovarian cancer cells [138]. Paclitaxel (PTX) combined with platinum was used as first‑line chemotherapy for ovarian cancer; however, the exact mechanism remains unclear [139]. Yang and colleagues discovered that after PTX treatment, the caspase-3/GSDME pathway was activated to disrupt cell membranes and induce tumor cells pyroptosis [140]. Bexarotene is a selective activator of the retinoid X receptor, commonly used against cutaneous T-cell lymphoma [141]. Interestingly, a study revealed that bexarotene treatment activated caspase-4 instead of caspase-3 and encouraged GSDME-dependent pyroptosis in ES2 cell line, indicating that bexarotene may be a novel agent for treating ovarian cancer [142].

### GSDME-mediated pyroptosis and other cancers

Glioblastoma multiforme is an aggressive, malignant, and fatal brain tumor that is refractory to the majority of therapeutic strategies [143]. As a second-generation cyclin-dependent kinase (CDK) inhibitor, AT7519 suppresses human glioblastoma cell growth via the caspase-3/GSDME**-**mediated pyroptotic pathway, suggesting that AT7519 is a potential GBM treatment chemical agent [144]. Kong and co-workers discovered that the natural flavonoid Galangin exhibits potent anti-glioblastoma multiforme properties [70]. GSDME knockdown in glioblastoma multiforme cells increased nuclear DNA damage and inhibited Galangin-induced pyroptosis [70].

Nasopharyngeal carcinoma is a malignant head and neck cancer type with high morbidity, characterized by an insidious onset and difficult early diagnosis [145]. Ovarian tumor family deubiquitinase 4 (OTUD4) has been found to be down-regulated in various types of tumors, and decreased OTUD4 expression is indicative of a poor prognosis [146]. A recent study demonstrated that OTUD4 deubiquitinated and stabilized GSDME, thereby promoting GSDME-mediated pyroptosis and increasing radiosensitivity in nasopharyngeal carcinoma [146]. Consequently, targeting the OTUD4/GSDME pathway to trigger pyroptosis is a novel strategy to improve radiotherapy sensitization of nasopharyngeal carcinoma [146]. Another study revealed that the natural product triptolide effectively eradicated head and neck cancer cells via GSDME-dependent pyroptosis by inhibiting mitochondrial hexokinase-ΙΙ [147].

As the most prevalent primary bone cancer, osteosarcoma has a high propensity for local invasion and metastasis [148]. Dioscin, a steroidal saponin extracted from medicinal plants, exhibits anti-inflammatory and anti-cancer properties [149]. A recent has confirmed that dioscin suppresses osteosarcoma cell proliferation via JNK/p38-dependent apoptotic pathway and GSDME-mediated pyroptotic pathway, implying that dioscin is a potential therapeutic drug for this disease [150].

Prostate cancer is the most prevalent type of tumor in males [151]. In a recent study, Zhang et al. synthesized a novel 3’,5’-diprenylated chalcone to develop new chemotherapy agents. They discovered that 3’,5’-diprenylated chalcone treatment initiates the PKCδ/JNK axis, thereby activating caspase-3 to cleave PARP and GSDME and trigger apoptosis and pyroptosis in prostate cancer cells [152].

## The role and mechanism of GSDME-mediated pyroptosis in tumor immunotherapy

The TIME plays a significant role in tumor formation, involving immune cells such as cytotoxic T lymphocytes (CTLs or CD8^+^ T cells), NK cells, and tumor-associated macrophages (TAMs) recruitment [153–155]. Increasing evidence implies that the activated state of the TIME can effectively eliminate tumor cells [156]. However, under specific conditions, TIME can aid tumor immune evasion and promote tumor growth [157].

In fact, GSDME-mediated pyroptosis has a dual effect on tumor immunotherapy. Firstly, prolonged exposure to the inflammatory TIME can stimulate tumor cell proliferation and metastasis [158]. However, GSDME overexpression can also considerably increase the quantity of NK cells and CD8^+^ cytotoxic T cells within tumors [21]. Granzyme B, derived from these immune cells, can directly cleave GSDME to initiate cancer cell pyroptosis, thereby transforming the TIME from a ‘noninflamed’ to an ‘inflamed’ state, which in turn increases immune cells recruitment and contributes to an improved response to immunotherapy [21]. The accumulating evidence indicates that therapeutic strategies aimed at inducing pyroptosis could trigger protective anti-tumor immune responses or expand immunotherapy responses to immune checkpoint inhibitors (ICIs) or other immunotherapies [159].

**Fig. 5:**
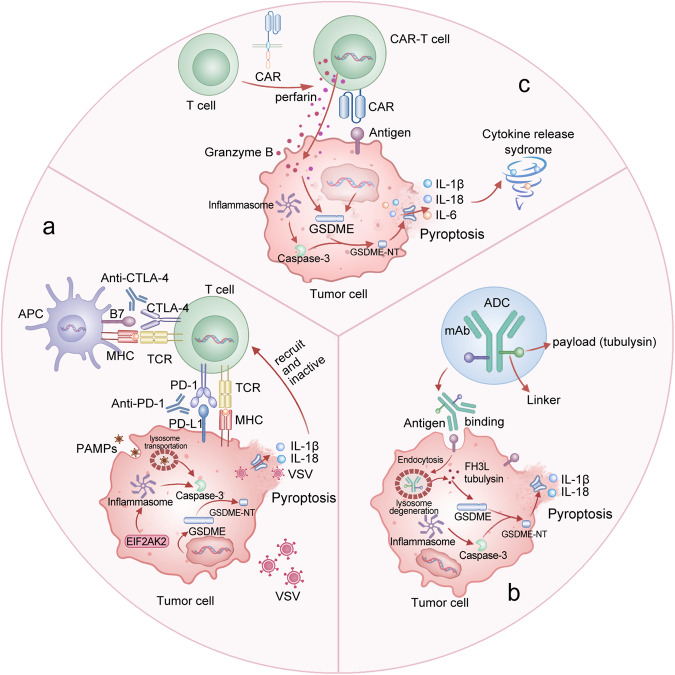
GSDME-mediated pyroptosis in anti-tumor immunotherapy: role and mechanism. **a** GSDME-mediated pyroptosis and Anti-PD-1 therapy. Expression of GSDME regulates polarity changes in TAMs, Tregs, and T cells. GSDME may modulate immune infiltration via EIF2AK2 and enhance the antitumor activity of anti-PD-1 therapy. Particularly, GSDME-mediated pyroptosis is crucial for the recruitment of cytotoxic T lymphocytes in the context of VSV therapy, which can transform immunologically “cold” tumors into “hot” tumors and enhance the efficacy of anti-PD-1 therapy. **b** GSDME-mediated pyroptosis and ADC therapy. ADCs recognize target cells by binding to cell surface antigens, resulting in internalization of the construct and payload release inside lysosomes through the internal environment or enzymes. Notably, tubulysin has been incorporated as a cytotoxic payload in ADCs; the binding of tubulysin and dendritic cells modulates Fms-like tyrosine kinase-3 ligand (Flt3L) restores antitumor immunity in GSDME-silenced tumors. **c** GSDME-mediated pyroptosis and CAR-T therapy. Granzyme B released by CAR-T cells entered tumor cells and induced pyroptosis by cleaving GSDME, which activated the GSDME-mediated pyroptotic pathway and triggered cytokine release syndrome.

It has been confirmed that GSDME-mediated pyroptosis plays a crucial role in anti-programmed cell death protein 1 (PD-1) therapy, antibody-drug conjugates (ADCs) therapy, and chimeric antigen receptor T cells (CAR-T cells) therapy [160–162] (Fig. 5).

### GSDME-mediated pyroptosis and Anti-PD-1 therapy

Immune evasion is a crucial feature of malignancies, as tumors tend to lose their immunogenicity and escape immune surveillance during division and proliferation. At present, ICIs exhibit promising potential in clinical oncology therapy [163]. ICIs bind to cytotoxic T-lymphocyte antigen-4 (CTLA-4) or PD-1, the primary targets associated with T-cell activation and exhaustion, and then eradicate tumor-induced immune suppression [164–166]. However, only one-third of patients respond to ICIs, thereby highlighting the urgent need to understand the mechanisms of resistance and identify suitable biomarkers that can accurately predict the clinical effectiveness of anti-PD-1 treatment [167]. The efficacy of anti-PD-1 therapy is closely related to TIME. Increasing evidence implicates the potential connection between anti-PD-1 treatment and GSDME-mediated pyroptotic cell death in tumors [168]. Hu et al. reported that GSDME expression could regulate polarity alterations in TAMs, Tregs, and T cell exhaustion [169]. GSDME might modulate immune infiltration through EIF2AK2 and augment the anti-tumor effect of anti-PD-1 therapy [169]. This study indicates that GSDME may be used as a predictive indicator of immune cell infiltration in various malignancies, including liver and lung cancers [169]. Oncolytic therapy is an emerging anti-tumor strategy that employs natural or engineered oncolytic viruses to inhibit the progression of tumors. According to a recent study, oncolytic vesicular stomatitis virus (VSV) activates GSDME-mediated pyroptosis to induce tumor cell pyroptosis [170]. Notably, GSDME is essential for recruiting cytotoxic T lymphocytes in the context of VSV therapy, which can transform immunologically ‘cool’ tumors into ‘hot’ tumors and increase the effectiveness of anti-PD-1 therapy [171]. Liang et al. designed a drug-polymer hybrid supramolecular nanoprodrug (PDNP) as a pyroptosis inducer to enhance tumor immunogenicity for an effective anti-tumor immune response [172]. In conjunction with anti-PD-1 therapy, PDNP induces GSDME-mediated pyroptosis and enhances the immune response in solid tumors, effectively halting invasive metastasis and extending survival due to its remarkable anti-tumor immunity [172].

Collectively, GSDME-mediated pyroptosis exhibits promise in augmenting anti-PD-1 treatment effectiveness and involves in tumor progression. In-depth research on this mechanism will open up novel perspectives for anti-tumor therapy.

### GSDME-mediated pyroptosis and antibody drug conjugate therapy

ADCs are a novel class of drugs that exploit the specificity of monoclonal antibody (mAb) to deliver a potent cytotoxic payload to target antigens expressed on tumor cells [173, 174]. ADC therapy occupies an increasing role in the arsenal of anticancer treatments [175, 176]. Recently, research has revealed that ADC treatment stimulates pyroptotic cell death and induces anti-tumor immunity in GSDME-expressing carcinomas [172]. The binding of ADC to dendritic cells modulates Fms-like tyrosine kinase-3 ligand (Flt3L) restores anti-tumor immunity in GSDME-silenced tumors and can potentially improve clinical outcomes [162]. In mouse models of colon and breast cancer, tubulysin, a microtubule depolymerizing agent, can trigger tumor cell death by targeting the GSDME-mediated pyroptotic pathway and modulating tumor sensitivity to ADC treatment [162].

Consequently, ADC therapy may induce GSDME-mediated pyroptotic cell death in GSDME-expressing cells, thereby regulating anti-tumor immunity and therapeutic response.

### GSDME-mediated pyroptosis and CAR-T therapy

Cancer immunotherapy has entered an era with the advent of CAR-T therapy [177]. Accelerated development of flexible and modular CAR-T systems enables multiple precise programming, antigen targeting, and adaptable solutions in cellular immunotherapy [178–180]. Due to their higher affinity for ligands compared to conventional T-cells for MHC peptide antigens, CAR-T cells have been engineered to enhance their killing ability by incorporating costimulatory domains on the receptor [181]. As a result, CAR-T cells are more effective in inducing pyroptosis compared to non-engineered CTLs [182]. Recent research indicates that CAR-T cells could activate the caspase-3/GSDME signaling pathway in B leukemia cells by releasing significant amounts of perforin and GzmB, which induce pyroptosis and enhance anti-tumor immunity [183]. Cytokine release syndrome (CRS), a consequence of extensive pyroptosis, is the most severe complication of CAR-T therapy [184]. Importantly, pyroptosis­related factors including IL-1β and IL-6 released by macrophages play a significant role in CRS-related inflammatory responses [185, 186]. Furthermore, GSDME elimination, macrophages removal, and caspase-1/GSDMD pathway inhibition could block CRS occurrence in mice. In addition, the severity of CRS is correlated with GSDME and lactate dehydrogenase levels in leukemia patients [183]. Notably, CAR-T cells release significantly more perforin/granzyme B than nontransduced natural T cells in order to cause target cell pyroptosis [187].

In summary, given the immunomodulatory effects of GSDME in TIME, GSDME-mediated pyroptosis exhibits the potential to improve CAR-T therapy efficacy. However, when utilizing CAR-T cells for malignancy treatment, the occurrence of CRS should also be considered. It might be a promising strategy to assess the risk and severity of CRS by detecting GSDME expression levels.

## Contribution Of GSDME-mediated pyroptosis to chemotherapy toxicity

Chemotherapy frequently causes severe toxic and side effects in cancer patients, which is one of the major limitations of chemotherapy medications in clinical cancer treatment [188]. Despite being silenced in most cancer cells, GSDME is expressed in many normal tissues, such as the brain, heart, gastrointestinal tract, and hematopoietic cells [49, 189–191]. Due to the fact that nearly all chemotherapy drugs lack tumor-specific GSDME targeting properties, toxicity and adverse effects are frequently induced during the treatment process [58, 192]. Recent research indicates that chemotherapy drugs elicit pyroptosis in human primary cells through the caspase-3/GSDME pathway [22]. While GSDME knockout significantly alleviated various tissue damage and weight loss caused by chemotherapy drugs including cisplatin, 5-Fluoruracil (5-Fu) and bleomycin in mice [193]. These findings introduce the novel concept that caspase-3 activation can induce GSDME-mediated pyroptosis and provide novel insights into cancer chemotherapy and toxic side effects [193]. In renal tubular epithelial cells, the depletion of GSDME inhibits pyroptosis induced by cisplatin, whereas 2-bromopalmitate (2-BP) suppresses chemotherapy-induced pyroptosis [81]. According to these findings, GSDME-targeted treatments may effectively overcome the nephrotoxicity associated with chemotherapy [81]. Clinical applications of doxorubicin have been hampered by severe cardiotoxicity during treatment [194]. Increasing evidence implicates that the abundant expression of GSDME in cardiomyocytes and generated mitochondrial ROS overflow may be associated with their mediated cardiotoxicity, implying that GSDME could be a potential target for mitigating the adverse effects of doxorubicin [195]. Hand-foot syndrome is a distinctive and frequent dermatological toxic effect of capecitabine-containing chemotherapy, but its exact mechanism remains unknown. Recent research indicates that it is possibly associated with elevated thymidine phosphorylase-mediated locoregional toxicity and disrupts keratinocytes via GSDME-driven pyroptosis, which induces robust and persistent inflammation [196]. It is noteworthy that macrophages also express GSDME. Chemotherapy-induced and GSDME-dependent pyroptosis of macrophages may not only contribute to the depletion of tissue-resident macrophages and impairment of innate immunity, but it may also engender a systemic inflammatory milieu that weakens the chemotherapeutic effects on cancers [197].

Overall, due to the fact that GSDME is typically methylated in tumor cells and expressed in most normal tissues, GSDME-mediated pyroptosis is a biological process responsible for the toxicity and side effects of certain chemotherapeutic medications (Table 2). Therefore, how to avoid this adverse effect caused by GSDME-mediated pyroptosis and provide tumor GSDME-specific targeted therapy is the focus of future research.

**Table 2:** GSDME-mediated pyroptosis contributes to chemotherapy toxicity.

Chemotherapy agents	Toxic side effects	Potential mechanism	Ref.
Cisplatin	Spleen lymphocytes decreased, neutrophil infiltration, vascular damages, acute kidney injury, and depletion of tissue-resident macrophages	Directly causes spleen lymphocytes, vascular endothelial cells, renal tubular epithelial cells, and macrophages injury via the caspase-3/GSDME pyroptosis pathway	[81, 193]
5-Fluoruracil (5-Fu)	Hemorrhage, inflammatory cell infiltration and loss of small intestine crypts	Causes toxicity to small intestine damage via the caspase-3/GSDME pyroptosis pathway	[193]
Bleomycin	Acute lung injury	Overactivate the caspase-3/GSDME-mediated pyroptosis in normal tissues	[193]
Doxorubicin	Cardiotoxicity	GSDME triggered pyroptosis in caspase-3 dependent reactions and generated mitochondrial ROS overflow in cardiomyocytes	[194, 195]
Capecitabine	Hand-foot syndrome	Elevated thymidine phosphorylase-mediated locoregional toxicity and disrupts keratinocytes through GSDME-driven pyroptosis	[196]

## Strategies to improve capacity to target GSDME and avoid side effects

Improper management of pyroptosis induction could result in detrimental effects on adjacent normal tissue to the tumor. Therefore, achieving a balance between tumor treatment and damage to healthy tissues is a critical issue that must be resolved. Tumor-targeting nanomaterials may be a potential strategy, which combines pyroptosis inducers and certain antibodies on the surface of nanoparticles and enhances tumor targeting capacity. Recently, Ding and colleagues reported that Pluronic F127-modified CCCP-incorporated ZIF-8 NPs (^F127^ZIF-8_CCCP_ NPs) induce tumor cell pyroptosis, thereby reprogramming the immunosuppressive tumor microenvironment and inhibiting tumor growth with high efficiency [198]. This facilitates a more favorable allocation of inducers within the TIME while reducing the adverse effects of pyroptosis activation [198]. Another study confirmed that MCPP, a nano-prodrug loaded with the phototoxic agent purpurin 18 (P18) and the cytotoxic agent PTX, exhibited an exceptional ability to induce GSDME-dependent pyroptosis with fewer damage to healthy tissues [199]. After administering MCPP to tumor cells and followed by laser irradiation, MCPP selectively triggered GSDME-dependent tumor cell pyroptosis and improved immune checkpoint blockade efficiency [199]. Wang et al. designed a self-supplying GSDME cooperative Nano-CRISPR scaffold (Nano-CD) intended for immunotherapy by promoting intracellular pyroptosis [200]. Mechanistically, the adjuvantic properties of the lytic intracellular content and the increased expression of GDSME facilitate cascade-amplification of the anti-tumor immune response [200]. This process ultimately leads to pyroptosis and tumor-associated antigens release [200]. Nowdays, outer membrane vesicles (OMVs) have emerged as a potential platform for the development of cancer immunotherapeutic agents and vaccines. Chen et al. developed molecularly engineered OMVs by equipping DNA aptamers on OMVs (Apt-OMVs) to facilitate secure and effective immunotherapy via targeting pyroptosis [201]. By acting as a natural carrier of LPS, Apt-OMVs stimulate cancer cell pyroptosis via the LPS-triggered pathway with high efficiency [201]. Consequently, selectively trigger GSDME-mediated pyroptotic tumor cell death could enhance tumor immunogenicity and avoid immune attacks and side effects.

## Conclusion and perspectives

The study of pyroptosis in tumors has become an extensive and rapidly developing field in recent years. The newly developed tumor pyroptosis therapeutic strategy possesses considerable promise. Most malignancies exhibit positive or negative regulation of GSDMs expression, which is associated with distinct tumor prognosis. Until now, the activation mechanism of GSDMs A–C and their physiological function in cancer remained poorly understood. GSDMD is the most well-studied GSDM; however, the effects of GSDMD-mediated pyroptosis on tumors are intricate. Elevated GSDMD expression predicts poor prognosis for lung and liver cancers, promoting immune escape of tumor cells [202, 203]. Conversely, it was discovered that GSDMD acts a tumor suppressor gene in colorectal and breast cancers [204, 205]. These findings suggest that GSDMD-mediated pyroptosis might exhibit tissue specificity and perform distinct functions in various organs and tissues. Compared to other GSDMs, GSDME is more likely to act as a tumor inhibitor via activating pyroptosis and correlate with anti-cancer immunity. Evidence shows that the absence of GSDME in tumors might trigger chemotherapy drug resistance. It is noteworthy that GSDME is epigenetically inactivated by promoter DNA methylation in most cancer lines and primary cancers, and its applicability as a marker makes it more favorable for further study than other GSDMs. Therefore, evaluating GSDME methylation levels is a beneficial strategy for early detection and distinguish between different tumor types. In fact, an emerging view is that targeting GSDME-mediated pyroptosis in localized tumors has a profound effect on immune cell recruitment in TIME and the immunotherapy response.

**Fig. 6:**
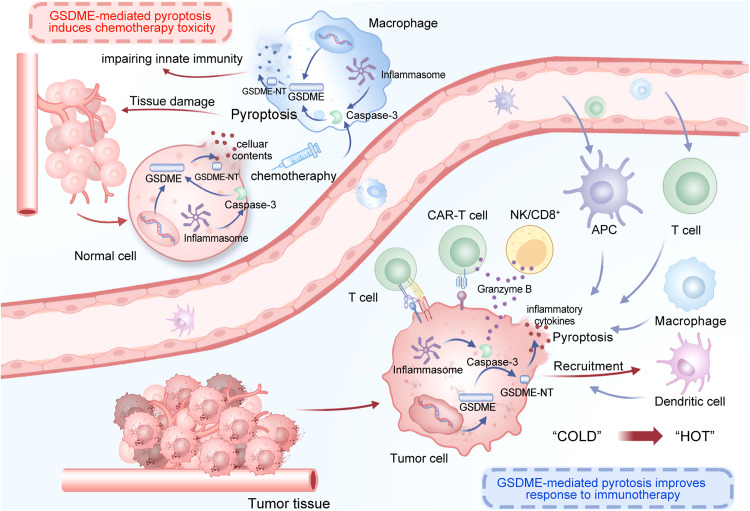
GSDME-mediated pyroptosis is a ‘double-edged sword’ in anticancer treatment. On one side, tumor suppression of GSDME involves inducing cancer cell pyroptosis and releasing inflammatory cytokines. These molecular events help convert TIME from a predominantly ‘cold’ state to a ‘hot’ state. As a result, the anti-tumor activity of CD8^+^ T killer lymphocytes, macrophages, and tumor-infiltrating NK cells is significantly enhanced. However, because GSDME is typically methylated in tumor cells and expressed in normal tissue cells and tumor-infiltrating macrophages, GSDME-mediated pyroptosis is a biological process responsible for certain chemotherapeutic medications’ toxicity and side effects.

In this review, we demonstrate extensive cross-talk between GSDME-mediated pyroptosis and anti-tumor immunity mechanisms based on existing laboratory and clinical evidence. As a potential tumor biomarker for early detection, diagnosis, prognosis, and treatment, GSDME methylation holds tremendous promise. Notably, in terms of anti-cancer treatment, GSDME-mediated pyroptosis is a ‘double-edged sword’ (Fig. 6). On the one hand, tumor suppression of GSDME is accomplished by cancer cell pyroptosis and inflammatory cytokines release, which transform the TIME from a ‘cold’ to a ‘hot’ state and considerably enhance the anti-tumor activity of CD8^+^ T killer lymphocytes, macrophages, and tumor-infiltrating NK cells. However, GSDME is also abundantly expressed in normal tissue cells and tumor-infiltrating macrophages, which can exacerbate chemotherapy toxicity and side effects. How to strike a balance between the two sides is an extremely critical research topic. Recent studies have identified tumor-targeting nanomaterials, photodynamic therapy, and Apt-OMVs as promising approaches to tackle these challenges by precisely targeting cancer cells. Furthermore, the role of GSDME-mediated pyroptosis in tumor immunotherapy has shown significant potential. Understanding its involvement in anti-PD-1 therapy, ADC therapy, and CAR T-cell therapy has provided innovative strategies for optimizing immunotherapy. In the near future, however, medical organizations are encouraged to conduct clinical trials in which patients are treated with approved medicines that regulate GSDME-mediated pyroptosis in conjunction with tumor immunotherapy.

Clearly, it is evident that GSDME-mediated pyroptosis has yet to disclose all its secrets involved in the pathologic process of cancer. Many questions are still open to be answered. If GSDME-mediated pyroptosis interacts with other signaling pathways to regulate tumor immunotherapy? Does detecting GSDME expression levels could be a promising strategy to assess the risk and severity of CRS caused by CAR T-cell therapy? What is the distinction between the spilled cellular contents of immune cells and cancer cells during GSDME-mediated pyroptosis? How different TIME cell types (e.g., tumor, stromal, and immune cells) interact to suppress or promote tumor progression through immunity or metabolic reprogramming? Would it be feasible if tumor-specific GSDME targeting therapy could prevent chemotherapeutic medications’ toxic effects in humans? In summary, a deeper understanding of the mechanisms underlying GSDME-mediated pyroptosis in TIME will aid in the development of novel and effective anti-cancer therapies. We believe that GSDME-focused research will yield novel insights into tumor diagnosis and treatment.

## Data Availability

All data generated or analyzed during this study are included in this published article.
